# Facial Affect Recognition by Patients with Schizophrenia Using Human Avatars

**DOI:** 10.3390/jcm10091904

**Published:** 2021-04-28

**Authors:** Nora I. Muros, Arturo S. García, Cristina Forner, Pablo López-Arcas, Guillermo Lahera, Roberto Rodriguez-Jimenez, Karen N. Nieto, José Miguel Latorre, Antonio Fernández-Caballero, Patricia Fernández-Sotos

**Affiliations:** 1 Servicio de Salud Mental, Complejo Hospitalario Universitario de Albacete, 02006 Albacete, Spain; nora.muros@gmail.com (N.I.M.); c.forner@hotmail.com (C.F.); knieto@sescam.jccm.es (K.N.N.); pfsotos@sescam.jccm.es (P.F.-S.); 2 Departamento de Sistemas Informáticos, Universidad de Castilla-La Mancha, 02071 Albacete, Spain; Arturosimon.Garcia@uclm.es; 3 Instituto de Investigación en Informática de Albacete, Universidad de Castilla-La Mancha, 02071 Albacete, Spain; 4 Servicio de Anestesiología, Reanimación y Unidad del Dolor, Hospital General de Villarrobledo, 02600 Villarobledo, Spain; plarcascalleja@gmail.com; 5 Departamento de Medicina y Especialidades Médicas, Universidad de Alcalá, 28805 Madrid, Spain; guillermo.lahera@gmail.com; 6 CIBERSAM (Biomedical Research Networking Centre in Mental Health), 28029 Madrid, Spain; roberto.rodriguez.jimenez@gmail.com; 7 Instituto de Investigación Sanitaria Hospital 12 de Octubre (imas12), 28041 Madrid, Spain; 8 CogPsy-Group, Universidad Complutense de Madrid, 28040 Madrid, Spain; 9 Departamento de Psicología, Universidad de Castilla-La Mancha, 02071 Albacete, Spain; Jose.Latorre@uclm.es

**Keywords:** schizophrenia, social cognition, emotion recognition, facial affect recognition, dynamic virtual humans

## Abstract

People with schizophrenia have difficulty recognizing the emotions in the facial expressions of others, which affects their social interaction and functioning in the community. Static stimuli such as photographs have been used traditionally to examine deficiencies in the recognition of emotions in patients with schizophrenia, which has been criticized by some authors for lacking the dynamism that real facial stimuli have. With the aim of overcoming these drawbacks, in recent years, the creation and validation of virtual humans has been developed. This work presents the results of a study that evaluated facial recognition of emotions through a new set of dynamic virtual humans previously designed by the research team, in patients diagnosed of schizophrenia. The study included 56 stable patients, compared with 56 healthy controls. Our results showed that patients with schizophrenia present a deficit in facial affect recognition, compared to healthy controls (average hit rate 71.6% for patients vs 90.0% for controls). Facial expressions with greater dynamism (compared to less dynamic ones), as well as those presented from frontal view (compared to profile view) were better recognized in both groups. Regarding clinical and sociodemographic variables, the number of hospitalizations throughout life did not correlate with recognition rates. There was also no correlation between functioning or quality of life and recognition. A trend showed a reduction in the emotional recognition rate as a result of increases in Positive and Negative Syndrome Scale (PANSS), being statistically significant for negative PANSS. Patients presented a learning effect during the progression of the task, slightly greater in comparison to the control group. This finding is relevant when designing training interventions for people with schizophrenia. Maintaining the attention of patients and getting them to improve in the proposed tasks is a challenge for today’s psychiatry.

## 1. Introduction

Schizophrenia is among the disorders with the greatest impact on a clinical, economic and social level [1]. Although its clinical manifestations are remarkably diverse, there are four cardinal symptomatic dimensions including the positive (hallucinations, delusions, and disorganized speech and behavior) and the negative dimension (including apathy, associability, anhedonia, alogy and emotional flattening), mood disturbances and cognitive deficits (including neurocognition and social cognition) [2]. According to current literature, negative and cognitive symptoms (and especially social cognitive deficits) seem to have a greater long-term influence on both quality of life and functioning [3,4]. Social cognition may be defined as those mental operations involved in social interactions, including the processes of perception, interpretation, and generation of responses to the intentions, dispositions and attitudes of others [3,5]. It is divided into four partially overlapping domains: social perception, attributional style, theory of mind and emotional processing [5,6].

In this article we focus on emotional processing, described as the aptitude to identify, facilitate, regulate, understand and handle emotions [7]. This domain is in turn divided into three subdomains that include lower and upper-level processes. The higher-level processes encompass understanding and emotional management while the lower perceptual level includes facial recognition of affect. This is described as the identification and recognition of emotional states through facial expressions and non-facial cues such as voice [5]. Facial recognition is a cornerstone in this process of perceiving the intentions and dispositions of others and then guiding social interactions [8].

In schizophrenia, impairments in the recognition of both faces and facial emotions have been documented [9]. It has been proposed that people with schizophrenia would not perceive faces holistically, but rather as a sum of parts and that the deficit would lie in this configuration analysis. For these patients, the greatest difficulty lies in recognizing negative emotions, like anger or fear, as well as the neutral emotion [10]. Deterioration seems to be fairly stable over the course of the disorder, unrelated to pharmacological treatment and irrespective of general cognitive deficits [11]. Some studies report that there is a correlation between the degree of impairment in facial recognition of emotions and the degree of psychopathology [12,13]. In turn, the deficit has been described in patients with first psychotic episodes and in subjects at significant risk of contracting psychosis, as well as in healthy relatives [10,11].

Different tools are available to evaluate facial emotion recognition. Most of these tools use a natural format, which has been criticized by different authors arguing that this type of stimulus does not represent the nature of the facial stimulus [14,15]. To overcome these limits, other studies have used videos to present more genuine expressions. However, these videos have not been validated and have several limitations in terms of length and scene format [16]. As far as computerized interventions are concerned, virtual reality (VR) has gained importance in recent years by providing practically real environments and situations [17,18,19], using dynamic avatars enabling social interaction with the participant which in turn may be manipulated to represent various emotional states [16,20,21]. VR enables the real-time assessment of individuals’ emotions, thoughts, behaviors and physiological responses in a built environment that can be controlled unlike a real environment [22]. Most of the studies related to the development of avatar faces take the Facial Action Coding System (FACS) as a reference. FACS encodes muscle contraction as a unit of measure called action unit (AU), helping to accurately catalog facial movements [23].

Our multidisciplinary research teamis developing a new facial emotion recognition intervention called “AFRONTA: Affect Recognition Through Avatars” aimed at improving facial affect recognition in patients with schizophrenia. To this end, a series of methodological steps are being taken prior to the implementation of the therapy. Firstly, a new collection of dynamic virtual faces (DVFs) representing the six basic emotions has been designed, precisely based on the AUs of the FACS system. The complete design process has been published accordingly [14]. Afterwards, these faces have been validated in 204 healthy people, in order to have a clear idea of the percentage of success in recognizing emotions shown in those faces [24]. The results showed that virtual humans were valid to recreate accurately the facial expressions of human-like emotions. The present article goes one step further, showing the results of the recognition scores of stable patients with a diagnosed schizophrenia and their comparison with a group of healthy controls. For this, considering previous articles, the following hypotheses are established:

**Hypothesis** **1** **(H1).** *Patients with schizophrenia will show a lower rate of emotional recognition and longer reaction times compared to healthy controls.*

**Hypothesis** **2** **(H2).** *Emotional recognition rates will be lower for negative and neutral emotions compared to positive emotions in the schizophrenia group.*

**Hypothesis** **3** **(H3).** *For both groups, more dynamic virtual faces (DVFs) will be recognized with a greater precision compared to the less dynamic ones, getting a higher number of successes.*

**Hypothesis** **4** **(H4).** *For both groups, faces presented in frontal view will be recognized with greater precision compared to faces presented in profile views, obtaining a greater number of hits.*

**Hypothesis** **5** **(H5).** *For the schizophrenia group, the number of hospitalizations throughout life will not influence successful emotional recognition.*

**Hypothesis** **6** **(H6).** *For the schizophrenia group, a relationship will be observed between the degree of psychopathology and emotional recognition.*

**Hypothesis** **7** **(H7).** *For the schizophrenia group, a relationship will be observed between the degree of functioning and quality of life and emotional recognition.*

**Hypothesis** **8** **(H8).** *For the schizophrenia group, there will be no significant differences regarding gender, age or education level.*

## 2. Materials and Methods

### 2.1. Study Design

The recruitment of patients with schizophrenia and healthy control participants, lasting 6 months (June to November 2020), occurred in the Mental Health Service of “Complejo Hospitalario Universitario de Albacete” (CHUA). The study was approved by the Clinical Research Ethics Committee of the hospital on 24 September 2019, with code number 2019/07/073. The Mental Health Service of CHUA serves a population of about 300,000 inhabitants.

### 2.2. Participants

The sample size was determined in 112 participants, including 56 stable patients diagnosed with schizophrenia and 56 healthy controls from the same demographic area. The sample size was established based on the number of stable patients who could be enrolled in the study. In accordance with a previous work [25], a patient was considered stable when he/she was on antipsychotic treatment and had been clinically stable (no hospital admissions, no changes in treatment, no significant psychopathological changes) for at least 3 months before inclusion. Patients were recruited consecutively by their treating psychiatrists after explaining the procedure and signing the informed consent. Healthy controls were enrolled in the same geographic area as the patients by request of the researchers from cultural centers and associations.

To ensure that the sample size provided an adequate level of statistical power, a sensitivity test was performed using the G*Power program (version 3.1.9.6) to calculate the minimum required effect size. A critical value d=0.640 was obtained for α=0.05, power = 0.95 (1−β), two sample groups with n=56, a non-centrality parameter δ=3.311, a critical value t=1.66 and 104.9 degrees of freedom. With respect to the effect size, what we have put at reference level is the critical value of Cohen’s *d*, which is indeed moderate. However, the equivalent effect size needs to be calculated for the various tests used, which are essentially non-parametric. Correspondences can be found on the website of Psychometrica [26].

The sociodemographic data of both patients and healthy controls are shown in Table 1. Gender and education level of both groups are exactly the same, because for each patient, a control was located with the same characteristics. This has resulted in age being very similar in both samples. The clinical data of the patients is described in Table 2.

Schizophrenia group:

All patients met DSM-5 diagnostic criteria for schizophrenia as measured by the Structured Clinical Interview for DSM-5 [22]. The mean of years of evolution of the disorder was 13.9 years (standard deviation, 9.7). The following criteria were established for the inclusion within the schizophrenia group:

(a) fulfilling diagnostic criteria DSM-5 for schizophrenia
(b) staying clinically stabilized during the 3 months prior to passing the semi-structured interview, according to criteria already used by our group [27]
(c) being an outpatient
(d) being aged between 18 and 65 years
(e) speaking fluent Spanish
(f) signing the informed consent form

On the other hand, the following exclusion criteria were considered:

(a) other axis I major mental disorders of DSM-5
(b) suffering mental retardation (intelligence quotient < 70)
(c) suffering somatic pathology that might interfere with facial affect recognition (for example, a significant visual impairment)

Healthy controls:

The only inclusion criterion for healthy controls was being between 18 and 65 years of age. Exclusion criteria comprised a mental illness diagnosis, a medical illness personal history (for example, a diabetic retinopathy affecting vision capacity) that could interfere with the study, and a first-degree family history of psychosis.

### 2.3. Data Collection Procedure

Schizophrenia group:

A screening evaluation was carried out by the referring psychiatrist to check if a patient met the inclusion/exclusion criteria during a clinical appointment. During the baseline visit, sociodemographic and clinical data were gathered, as well as personal psychiatric history, psychiatric family history, medical comorbidity, substance use (current and past), treatment (pharmacological and psychosocial), previous hospitalizations and visits to the emergency room. Before conducting the experiment, and after a careful explanation of the study, each patient had to sign an informed consent. The acquired data were stored anonymously using dissociated databases.

The sociodemographic data included: age, gender, race, civil status, place of residence (rural, urban), level of education (low, medium, high according to INE, the Spanish National Institute of Statistics) [14], employment status (active, unemployed, temporary incapacity for work, pensioner, housekeeper, student), and profession. Low education stands for lower secondary education and below (6–8 years primary); medium education includes upper secondary education and post-secondary non-higher education (12–14 years secondary); high education encloses university education (8–20 years tertiary or university education).

The clinical data included personal somatic history (including neurological), toxic personal history, psychiatric personal history (including diagnosis, years disease evolution, number of decompensations, number of hospitalizations), current treatment, time and dose and relevant family history. The variables and measurement instruments were:

- Spanish version [28] of the Positive and Negative Syndrome Scale, PANSS [29]. PANSS is a 30-item scale that provides a measure of positive symptoms, negative symptoms, and general psychopathology.
- The Functioning Assessment Short Test, FAST [30]. The 24 items of the FAST scale are divided among six specific areas of functioning: autonomy, occupational functioning, cognitive functioning, financial issues, interpersonal relationships and leisure time. A recent study noted that the FAST showed strong psychometric properties and was a valid instrument for use in clinical practice, clinical trials, and research settings in subjects diagnosed with schizophrenia [31].
- Spanish version [32] of the WHOQOL-BREF [33] assessment tool. WHOQOL-BREF is a generic questionnaire developed by the Study Group on Quality of Life of the World Health Organization which introduces a total of 26 questions to measure the quality of life. The scale is divided into four domains: physical, psychological, social relationships and environmental health.

Healthy controls:

Healthy controls were recruited in the same sociocultural living area where the schizophrenia group lived, mainly from similar cultural and social groups. The research team designed a data collection notebook that included sociodemographic and clinical data.

Clinical data comprised personal somatic (including neurological), personal toxicity and psychiatric, and pertinent relatives’ history. Data gathering was accomplished in a sole 30-min individual session. When the participant satisfied the inclusion criteria of the study, the facial stimulus was administered.

### 2.4. Experimental Procedure

**Figure 1:**
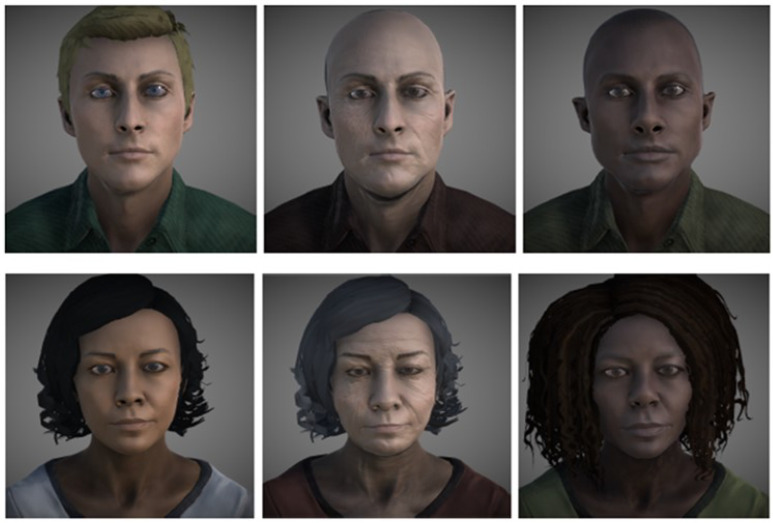
Examples of virtual faces.

All participants received 52 dynamic virtual faces (DVFs) presented on a 27” computer monitor. Some examples of DVFs are shown in Figure 1. The complete description of the DVFs employed can be consulted in our previous experiment that validated the DVFs in 204 healthy people [24]. The software tool and the DVFs developed for this research are publicly accessible at Universidad de Castilla-La Mancha institutional repository RUIdeRA at hdl.handle.net/10578/27021, accessed on 20 April 2021.

**Figure 2:**
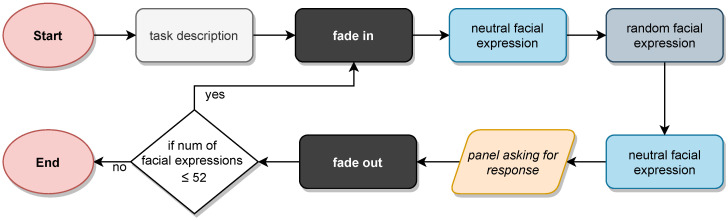
Outline of the experimental procedure.

The experiment was performed in one session of approximately 10–20 min. The experimental procedure is outlined in Figure 2. Firstly, a short tutorial introduced the participants to the task they had to perform. Then, the emotion identification test started and 52 DVFs were presented to them depicting the different emotions. In essence, they were to identify all the basic emotions presented (joy, sadness, anger, fear, disgust and surprise) plus neutral expression. These emotions were presented randomly to each participant. Each emotion appeared 8 times (with two levels of dynamism) plus 4 times the neutral expression. The virtual characters were faded-in from a black background. After that, they exhibited an emotion starting from and ending in the neutral expression, for a 2-s total exposure time. Participants had to label each expression conveyed by a DVF from among the offered seven alternatives. Among those 52 faces, 50% were displayed with less dynamism (only the most characteristic facial features of each emotion included movement) and another 50% showed more dynamic faces (additional added neck and shoulder movements to add realism and naturalness). In addition, the DVFs were shown by 50% in the frontal view, 25% in the right lateral face, and 25% in the left lateral face. Once each face was presented to the participants, a panel appeared on screen allowing them to click on the name of the expression they had just seen. When an option was selected, the character faded-out to a black background and the whole process started over again, repeating a total of 52 times.

The virtual characters depicting the emotions were also randomized. The DVF set comprised 2 Caucasian avatars in their 30 s, with varying features in terms of eye color, skin tone, and hair. In addition, 2 African race avatars in their 30 s and 2 elderly avatars were presented. Out of the 52 avatars featured, 8 were of African race and 8 were elderly.

### 2.5. Statistical Analysis

The IBM SPSS Statistics program (version 24) was used to perform the statistical analyses. Mean and standard deviation were employed to describe quantitative variables, whereas percentages were used for qualitative ones. Because the data (successes and reaction time) did not conform to a normal distribution, non-parametric methods were primarily used to test the hypotheses. *p*-Value < 0.05 was considered to be statistically significant. The Kruskal–Wallis test was used to find statistically significant differences in emotion recognition and reaction time between more than two groups of participants (i.e., the influence of age and education). When differences were found, the Dunn’s post-hoc test together with a Bonferroni correction for pair-wise comparisons was applied in order to find out which groups were different. The Mann–Whitney test was used when only two groups had to be compared. The Wilcoxon signed-rank test was used whenever any differences in the performance of the same group of participants using two techniques (i.e., DVF with higher vs. lower dynamism) were to be found. Associations among variables were analyzed using Spearman’s rank correlation coefficients.

## 3. Results

### 3.1. Comparison of Recognition Scores and Reaction Times of Healthy Controls and Patients with Schizophrenia

#### 3.1.1. Recognition Scores and Reaction Times in the Schizophrenia Group

A statistically significant correlation was found between the recognition scores and the reaction times (R=−0.561, p<0.001) for patients with schizophrenia. The negative correlation indicates that the patients who take the longest to answer do worse. This finding is in line with another study that has analyzed the relationship between reaction times and recognition rates in schizophrenia patients [34].

#### 3.1.2. Recognition Scores

The results for the recognition scores for healthy controls and patients are summarized in Table 3. The average score for healthy controls is 90.0%, while it is 71.6% for the schizophrenia group. This confirms hypothesis H1 in the sense that patients with schizophrenia show a lower rate of emotional recognition compared to healthy controls. The Mann–Whitney U Test confirmed this difference in the results to be statistically significant (U=415.0, p<0.001,d=1.64). Other differences become apparent when looking at the results for the individual emotions. The biggest differences are in fear, with a score of 45.5% for the schizophrenia group and 76.6% for healthy controls; disgust, 61.4% and 88.4%; joy, 76.8% and 97.1%; and sadness, 60.3% and 85.3%. However, statistically significant differences are also found in neutral (U=1275.0, p=0.011) and anger (U=1086.5, p=0.002), that join fear (U=623.5, p<0.001), disgust (U=655.0, p<0.001), joy (U=840.5, p<0.001) and sadness (U=655.5, p<0.001). In all cases, the score is higher for the control group.

In regard to hypothesis H2 (emotional recognition lower for negative and neutral emotions compared to positive emotions in the schizophrenia group), after studying negative, neutral and positive emotions, we can affirm that this is partially fulfilled. Negative emotions were recognized much better (82.3% hit rate) than negative ones (62.6%).

**Figure 3:**
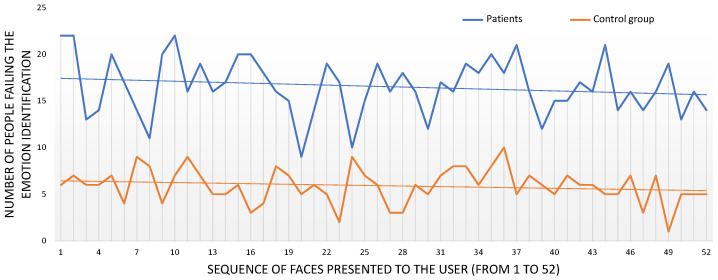
Emotion identification errors (y-axis). The x-axis refers to the sequence of 52 faces presented to the participants. The trend line shows a negative slope of 3.4% for the schizophrenia group and 2.1% for the control group.

In addition, we studied the evolution of the test to see whether the participants became tired and their emotion recognition rates worsened as the test progressed as a result, or whether they learned during the test and improved their recognition rates. For this, we plotted the number of emotion recognition errors for each of the 52 faces presented to the users (see Figure 3). It is worth noting that the order in which the emotions were presented was random, so it varied from participant to participant. Since we are studying the evolution of recognition rates, the presentation order is not relevant. This graph clearly shows a difference in the emotion recognition between healthy controls and patients, as it has already been identified. Moreover, the trend lines for each group have also been added to the graph. The slopes of these trend lines show a slight reduction in the number of errors as the test progressed, a reduction of 2.1% for the healthy controls and 3.4% for the schizophrenia group.

#### 3.1.3. Reaction Times

The time it took for healthy controls and patients to select on the emotions among the different alternatives was recorded for each face presented. We used the Mann–Whitney U test to investigate the differences in reaction time between the control and patient groups. There were statistically significant differences in the average reaction time (U=415.5, p<0.001,d=1.64) as well as for each individual emotion (U=633.0, p<0.001 for Neutral, U=562.0, p<0.001 for Surprise, U=402.0, p<0.001 for Fear, U=571.0, p<0.001 for Anger, U=654.0, p<0.001 for Disgust, U=435.5, p<0.001 for Joy and U=663.0, p<0.001 for Sadness). In all cases, the reaction time was lower for the control group, confirming that patients with schizophrenia show longer reaction times compared to healthy controls, as proposed under hypothesis H1.

**Figure 4:**
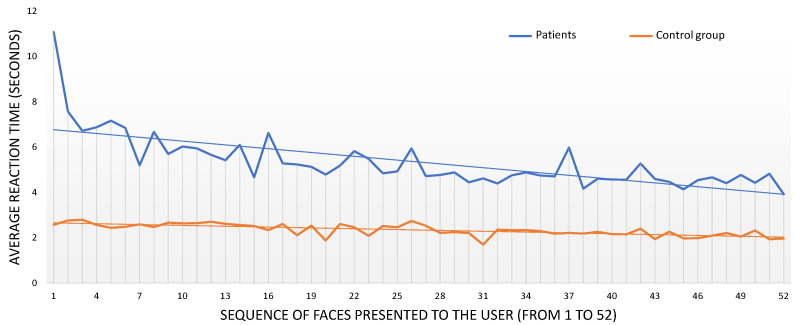
Average reaction time for each face presented to the users (y-axis). The x-axis refers to the sequence of 52 faces presented to the participants. The trend line shows a negative slope of 5.6% for the schizophrenia group and 1.2% for the control group.

We also studied reaction time as the test progressed. Figure 4 depicts the average reaction time obtained for the healthy controls (orange line) and the patients (blue line) as the test progressed (from the 1st to the 52nd face presented). As already mentioned, the presentation order of the faces differed from one participant to another, but it was not important when focusing on overall reaction time. Trend lines are also included in this graph, and the slopes of these trend lines reveal a reduction of 1.2% in the healthy controls reaction time and a reduction of 5.6% in the schizophrenia group. The reduction is more apparent for the patients, especially for the few first faces presented.

### 3.2. Influence of Dynamism and Presentation Angle of the DVFs on Emotion Recognition

#### 3.2.1. Dynamism of the DVFs

Table 4 shows the recognition scores of both schizophrenia patients and healthy controls with and without high dynamism on the DVFs. Regarding the impact of dynamism on emotion recognition for the schizophrenia group, the overall recognition rates when more dynamic DVFs were used is 72.5%, while it drops to 65.9% with fewer dynamic DVFs. The Wilcoxon Signed Ranks test found statistically significant differences in the overall emotion recognition scores (Z=−3.49, p<0.001, d=0.70) and in disgust (Z=−3.60, p<0.001) and joy (Z=−2.00, p=0.045). In all cases, the emotion recognition rates are significantly higher when more dynamic DVFs were used. Thus, as proposed by hypothesis H3, more dynamic virtual faces were recognized with greater precision compared to the less dynamic ones, reaching a higher number of successes, for both groups.

A lower reduction in the recognition scores is observed for healthy controls, with 90.7% for high dynamism and 87.1% for low dynamism. Similarly to the schizophrenia group case, the Wilcoxon Signed Ranks test found statistically significant differences (Z=−2.17, p=0.030). In this case, no other significant difference was found in the individual emotions.

We also compared the results for the schizophrenia group and healthy controls for high and low dynamism. For the former, statistically significant differences were found for the overall emotion recognition (U=2527.0, p<0.001), fear (U=2217.5, p<0.001), anger (U=1966.0, p=0.018), disgust (U=1903.5, p=0.046) and sadness (U=2301.5, p<0.001). In all cases, the results for healthy controls were higher than the schizophrenia group. For the latter, differences were found for the overall emotion recognition (U=2685.5, p<0.001), fear (U=2361.0, p<0.001), disgust (U=2372.0, p<0.001), joy (U=2259.5, p<0.001) and sadness (U=1905.0, p=0.046). Again, the results were significantly higher for the control group.

#### 3.2.2. Presentation Angle of the DVFs

Table 5 shows the recognition scores for the different presentation angles for both the schizophrenia group and healthy controls. A slight improvement in the recognition rates for frontal views is noticeable (72.6% for frontal and 70.5% for profile views) in line with hypothesis H4, which proposed that faces presented in frontal view would be recognized with greater precision compared to faces presented in profile views for both groups. However, the Wilcoxon Signed Ranks test could not find significant differences in the data for the overall recognition rates (Z=−0.53, p=0.600). Extending the study to individual emotions, Fear is the only one in which significant differences were found (U=−2.10, p=0.036), being the recognition rates higher for frontal views.

The impact of the presentation view seems lower for the control group (90.3% and 89.7% for frontal and profile views, respectively). This is confirmed by the Wilcoxon Signed Ranks test (Z=−0.54, p=0.591). The study of the differences in the individual emotions could not find statistically significant differences either.

The comparison of the results for the schizophrenia group and healthy controls by the presentation angle using the Mann–Whitney test revealed differences for the overall recognition rates for frontal views (U=487.5, p<0.001) and for profile views (U=468.0, p<0.001). In both cases, the emotion recognition is higher for the healthy controls than the schizophrenia group. A look at the individual emotions revealed differences in neutral (U=1347.0, p=0.025), fear (U=904.5, p<0.001), anger (U=1148.5, p=0.003), disgust (U=773.5, p<0.001), joy (U=1047.0, p<0.001) and sadness (U=487.5, p<0.001) for the frontal views, and in fear (U=622.0, p<0.001), anger (U=1270.5, p=0.026), disgust (U=845.5, p<0.001), joy (U=871.5, p<0.001) and sadness (U=468.0, p<0.001). In all cases, the results for the frontal views are higher than those for the profile views.

### 3.3. Influence of Sociodemographic Data on Emotion Recognition for the Schizophrenia Group

The results concerning gender, age and education level will be described next. As can be seen, hypothesis H8 was fulfilled, as no significant differences regarding gender, age or education level were detected.

#### 3.3.1. Influence of Gender

The influence of gender on emotion recognition was also studied. Regarding the schizophrenia group, the Mann–Whitney U test could not find significant differences for the overall recognition rates (U=292.0, p=0.244). Similar results were obtained for individual emotions but for Disgust (U=239.5, p=0.037), for which the correct identification rates were slightly higher for women than for men.

#### 3.3.2. Influence of Age

Several studies on facial emotion recognition in a healthy population have been conducted using two groups of participants: young (20–59 years) and old (≥60 years) [35]. It is also true that some study found differences between young adults (20–39 years) and older adults (40–59 years) in favor of the younger group [36]. Therefore, and in line with a recent paper by the same authors validating the set of avatars in healthy population [24], three age groups were established for the analysis: 18–39 years, 40–59 years and above (here, 60–65 years old).

Regarding the schizophrenia group, the Kruskal–Wallis test was used to find statistically significant differences in the number of correct identification of the emotion presented per group. This test could not find significant differences in the overall emotion recognition results ($\chi_{\left(2\right)}^{2}=4.07,\;p=0.131$). However, significant results were found for Neutral ($\chi_{\left(2\right)}^{2}=6.30,\;p=0.043$), and the post-hoc test revealed that the differences were between groups 18–39 and 60–65 (p=0.036), being the number of faces for which the emotion was correctly recognized higher for the group 18–39.

#### 3.3.3. Influence of Education Level

Statistically significant differences were found in the overall correct identification rates for the schizophrenia group ($\chi_{\left(2\right)}^{2}=14.92,\;p=0.001$). The post-hoc test discovered that those differences were in the results for a low education level when compared to medium (p=0.016) and high (p<0.001) education levels, being lower for the lower levels. Differences were also found for neutral ($\chi_{\left(2\right)}^{2}=11.08,\;p=0.004$), disgust ($\chi_{\left(2\right)}^{2}=9.23$, p=0.010) and Joy ($\chi_{\left(2\right)}^{2}=11.54,\;p=0.003$). For neutral, the post-hoc test revealed differences between low and medium education levels (p=0.005), with respect to the high education level group (p=0.014). For disgust, differences were between low and high levels (p=0.009). Finally, for joy the differences were between low and medium (p=0.044), with respect to the high (p=0.002) level.

#### 3.3.4. Influence of Being Active

No significant differences were found in the results for emotion recognition comparing patients with schizophrenia that are active (working or studying) or inactive (unemployed or retired) (U=176.0, p=0.140).

### 3.4. Influence of the Number of Hospitalizations and the Dosage of Antipsychotic Drugs on Emotion Recognition for the Schizophrenia Group

No correlation could be found for emotion recognition using Spearman’s rank correlation coefficient for the number of hospitalizations and the emotion recognition rate (R=−0.263,p=0.050) as expected from hypothesis H5 (for the schizophrenia group, the number of hospitalizations throughout life will not influence successful emotional recognition). Moreover, no correlation was found between the dosage of antipsychotic drugs and the facial emotion recognition rates (R=−0.197,p=0.145).

### 3.5. Influence of the Results of the Schizophrenia Group’s Psychometric Scales on Emotion Recognition

The results of the administration of the three different scales to the schizophrenia group is summarized in this section.

#### 3.5.1. Positive and Negative Syndrome Scale (PANSS)

The results of the PANSS scale are summarized in three different categories: Positive and Negative (whose results range from 7 to 49), and General Psychopathology (from 16 to 112). We analyzed the emotion recognition results based on Spearman’s correlation coefficient. We could not find statistically significant correlations between emotion recognition and the PANSS Positive (R=−0.207, p=0.125) and PANSS General Psychopathology (R=−0.066, p=0.627) scales. However, Spearman’s correlation coefficient has shown statistical significance in facial emotion recognition for the PANSS Negative scale (R=−0.378, p=0.004). After detecting such a meaningful correlation between negative symptoms and emotion recognition impairment, we tested the relationship between the recognition rates for positive, negative and neutral emotions, and the results of the PANSS Negative scale. There was no relation with the recognition rates of positive emotions (R=−0.009, p=0.949), but a negative correlation was found with the recognition rates of negative emotions (R=−0.426, p=0.001) as well as the neutral one (R=−0.337, p=0.11). In this regard, hypothesis H6, proposing that a relationship would be observed between the degree of psychopathology and emotional recognition, was fulfilled.

#### 3.5.2. Functional Assessment Short Test (FAST)

The result of the FAST scale can be summarized in a value that ranges from 0 to 72, 0 meaning the best functioning of the patient and 72, the worst. No statistically significant correlation was found between FAST and the recognition rates (R=0.112, p=0.411). This confirms that the number of emotions recognized is not influenced by the result of the FAST scale, as there is no reduction or increase in the number of emotions recognized for the different values of the FAST score. In terms of hypothesis H7, FAST did not confirm that a relationship would be observed between the degree of functioning and quality of life and emotional recognition.

#### 3.5.3. World Health Organization Quality of Life Field Trial Version (WHOQOL-BREF)

The results of the WHOQOL-BREF scale are provided in four domains: physical health, psychological, social relationships and the environment. The scores for each category range from 4 to 20. Using Spearman’s rank correlation coefficient, no statistical significance could be found in emotion recognition: physical health (R=−0.063, p=0.647), Psychological (R=−0.141, p=0.299), social relationships (R=−0.070, p=0.605), and the environment (R=−0.031, p=0.815). Similaily to FAST, WHOQOL-BREF could not confirm that a relationship exists between the degree of functioning and quality of life and emotional recognition (hypothesis H7).

## 4. Discussion

Consistent with hypothesis H1, results show that patients with schizophrenia have lower recognition hits on virtual emotional expressions when compared with the control group. This finding was observed for all emotions included. This result is congruent with those presented in previous studies using both natural and virtual stimuli. The schizophrenia group had longer reaction times, compared to healthy controls.

Facial emotion recognition impairment in schizophrenia has been known to be a solid finding. Although some authors pointed out the possibility that the use of dynamic avatars could improve successful recognition in patients with schizophrenia [37], deficits in facial emotion recognition appear to expand for the emotions expressed by VHs [13,38,39,40]. It should be noted that, compared with similar studies using VHs, the general precision in the identification of emotions was higher or equivalent both in the group of healthy controls (90.0%) and patients with schizophrenia (71.6%). For health controls the precision is 62.2% [41], 72.0% [42], 73.2% [43], 82.5% [16] and 91.0% [44]. In the case of patients with schizophrenia our scores outperform previous identification rates that go from 48.0% [13] to 60% [39]. Our results are consistent with previous studies on facial emotion recognition in patients with schizophrenia using emotion photographs. A meta-analysis that included 112 studies [45], showed a large effect for emotion processing (Hedges’ g=0.88). The effect size in early-onset and first-episode psychosis was very similar with the magnitude of impairment found to be large for disgust, fear and surprise, and medium for sadness and happiness [46]. Our new set of dynamic virtual faces shows similar differences but provides more measurement options and higher ecological validity.

With regard to reaction times, both groups presented a learning effect during the progression of the task, which was slightly greater for the schizophrenia group (both in terms of the number of hits and in the reduction of reaction times). This finding is tremendously relevant when designing training interventions for patients with schizophrenia. Maintaining patients’ attention and getting them to improve in the proposed tasks is a challenge for today’s psychiatry. In this first section regarding the rate of emotional recognition by patients with schizophrenia, we could conclude that the development of increasingly sophisticated VHs promotes successful emotional recognition, being superior to the use of static images. The use of valid ecological environments will allow simulating social interactions like reality, allowing therapists to control and manipulate the behavior of the avatars to evaluate and train basic emotional recognition.

As further expected from hypothesis H2, the differences between groups were more pronounced for some negative emotions (fear and disgust), a finding observed both in previous studies with both natural [47,48] and virtual stimuli [13,39]. Among the schizophrenia group, the worst recognized emotions were three of the four negative emotions presented, including disgust, sadness and fear (with success rates of 61.4%, 60.3% and 45.5%, respectively), while the best-recognized emotions were the positive ones, neutral emotion and anger. The high hit rate for neutral emotion (86.2%) and anger (83.3%), is striking. In the case of neutral emotion, the result can be explained (at least in part) by the dynamic characteristics that the avatars presented. While for the rest of the emotions the participant observed a transition from the neutral emotion to the final emotion (which involved a series of facial movements), this did not happen with the neutral emotion, in which the avatar only blinked.

As hypothesized under hypotheses H3 and H4, in both groups, the more dynamic DVFs and those ones presented in frontal view were recognized with greater precision. From our point of view, this is the first study that presents avatars with frontal and profile views to patients with schizophrenia. A study carried out by our research team in healthy controls also obtained better rates of emotional recognition when the avatars were presented from the frontal view, compared to the profile view [14].

As assumed from hypothesis H5, the number of hospitalizations throughout life was not correlated with successful emotional recognition. A meta-analysis on facial recognition of emotions in schizophrenia showed that the total number of past and present hospitalizations of the schizophrenia group did not appear to have a significant impact on facial recognition emotion [47].

Regarding the relationship between recognition deficits and the degree of psychopathology, a statistically significant correlation was found for the negative subscale of the PANSS, as proposed by hypothesis H6. More concretely, a negative correlation was found with the recognition rates of negative and neutral emotions. This finding supports the results obtained in the previous studies [13,39,47]. This could suggest that prefrontal hypodopaminergia associated with negative symptoms could also be related not only to neurocognitive deficits, but also to facial recognition of emotions and other domains of social cognition. In neuroimaging studies with subjects with schizophrenia, inferring the emotions of others elicited decreased activation in an extensive cluster incorporating the striatum, thalamus and amygdala, as well as in a large area of the dorsolateral and ventrolateral prefrontal cortex [49].

Contrary to expectations expressed under hypothesis H7, we did not find a significant correlation between the degree of functioning (evaluated through the FAST scale) or quality of life (measured through the WHOQOL-BREF scale) and emotion recognition. We also did not find a significant correlation with respect to employment status (active vs unemployed) and the recognition rate. Previous studies indicate the relationships between social cognition, functionality and quality of life in patients with schizophrenia [50]. Neurocognition and other domains of social cognition that are closely related to functionality and quality of life were not evaluated at work, which is likely to have influenced the results. Future jobs should include these types of evaluations.

As expected from hypothesis H8, no statistically significant differences were found in terms of gender, except for disgust, in favor of women, in the schizophrenia group. This same finding was obtained in a previous study conducted by our research team for healthy controls [14]. No gender differences were observed for the group of healthy controls. In the schizophrenia group, no significant differences were found in terms of age, except for the neutral emotion in favor of the younger group; in the control group statistically significant differences were found for the overall emotion recognition results, in favor of the younger group. We must point out that the study included participants up to 65 years of age, age from which a greater deterioration in facial recognition of emotions has been described, so the results in this regard are limited. New studies that include participants aged 65 and over will shed light on this. Contrary to what was hypothesized, significant differences were found with respect to education level for the group of patients, with lower recognition for patients with low education level compared to patients with medium and high levels. A meta-analysis on facial recognition of emotions in schizophrenia did not find differences in terms of education level [47].

The present study has some strengths and weaknesses. Among the weaknesses, it should be mentioned that nicotine habits within the groups has not been evaluated. Indeed, it has been established that nicotine influences a wide variety of cognitive domains [51,52]. It would also be interesting to try out a multivariate approach that could detect if there is a multivariate pattern that distinguishes patients with schizophrenia from controls, although this is beyond the scope of this paper. Another important issue is widening the range of emotions to non-basic ones and the number of DVFs available. The most important strength is related to face presentation views and the levels of dynamism of the DVFs, which has enabled studying some aspects that are usually not explored. Let us also highlight that it is the first time that this particular set of DVFs has been used on a clinical sample of patients with schizophrenia and the results have been compared with healthy volunteers.

## 5. Conclusions

This article has faced the difficulty of facial emotion recognition inherent to schizophrenia patients. Similarly to other recent approaches, we have created a new set of virtual human faces aimed at remediating this kind of social cognition deficit. Prior to using the human avatars in a novel facial emotion recognition intervention named “AFRONTA: Affect Recognition Through Avatars”, this work has evaluated the recognition rates with a schizophrenia group and a control group, which has enabled adequate discrimination between both groups.

This set of virtual faces already studied on healthy volunteers has also shown a significant ability to discriminate emotions by patients with schizophrenia, although with a lower hit rate than the healthy ones. The possibility of choosing the age, gender and ethnicity of the avatars opens up new possibilities for the introduction of new features in facial emotion recognition tests, which is not feasible with more traditional photo- and video-based solutions. Moreover, the capabilities of computerized systems make it possible to offer different views and levels of dynamism of the DVFs.

## Figures and Tables

**Table 1:** Sociodemographic data of the samples.

	Schizophrenia Group (n=56)	Control Group (n=56)
Age	38.0 ± 9.4	36.9 ± 11.1
Gender	Men: 35.7%	Men: 35.7%
	Women: 64.3%	Women: 64.3%
Education level	Basic: 21.4%	Basic: 21.4%
	Medium: 53.6%	Medium: 53.6%
	High: 25.0%	High: 25.0%

**Table 2:** Clinical data of the samples.

Patients treated with atypical antipsychotics only	55 (98.2%)
Patients treated with typical antipsychotics only	0 (0.0%)
Patients treated with atypical and typical antipsychotics	1 (1.8%)
Chlorpromazine equivalents	380 ± 53.6 mg/day
Age of onset of the disorder	23.71 ± 6.05 years
Years of disorder evolution	13.93 ± 9.66 years

**Table 3:** Emotion recognition scores (%) for each emotion depicted for the schizophrenia group and healthy controls.

Schizophrenia Group (n=56)								Control Group (n=56)						
	**Neutral**	**Surprise**	**Fear**	**Anger**	**Disgust**	**Joy**	**Sadness**	**Neutral**	**Surprise**	**Fear**	**Anger**	**Disgust**	**Joy**	**Sadness**
Neutral	86.2	0.9	2.2	1.8	1.8	3.6	3.6	97.3	0.0	0.0	0.0	0.9	0.0	1.8
Surprise	2.0	87.7	5.1	0.4	1.1	2.2	1.3	0.4	91.3	8.0	0.0	0.2	0.0	0.0
Fear	5.6	36.2	45.5	1.1	3.1	0.7	7.8	0.7	14.5	76.6	0.2	0.7	0.0	7.4
Anger	3.3	1.6	3.1	83.3	5.1	0.7	2.9	0.4	0.7	1.3	94.2	2.5	0.0	0.9
Disgust	4.5	2.2	3.8	23.9	61.4	0.4	3.8	0.4	1.1	1.6	8.5	88.4	0.0	0.0
Joy	10.3	4.7	1.1	2.5	3.3	76.8	1.3	2.0	0.9	0.0	0.0	0.0	97.1	0.0
Sadness	11.6	6.7	8.3	5.1	6.7	1.3	60.3	3.1	2.7	4.5	0.4	3.6	0.4	85.3

Notes: Columns: emotions recognized. Rows: emotions presented.

**Table 4:** Confusion matrix of emotion recognition (%) using high and low dynamism in DVFs when displaying emotions for the schizophrenia group.

**High Dynamism**														
**Schizophrenia Group (n=56)**								**Control Group (n=56)**						
	**Surprise**	**Fear**	**Anger**	**Disgust**	**Joy**	**Sadness**		**Surprise**	**Fear**	**Anger**	**Disgust**	**Joy**	**Sadness**	
Surprise	86.8	6.2	0.9	1.3	2.2	0.4		87.1	12.0	0.0	0.4	0.0	0.0	
Fear	38.3	52.8	0.5	2.3	0.5	2.8		16.0	79.9	0.5	0.9	0.0	1.8	
Anger	1.9	2.8	84.3	4.6	0.9	3.2		0.4	0.9	96.5	0.9	0.0	0.4	
Disgust	4.0	6.3	6.7	75.0	0.4	3.1		0.5	2.3	1.9	94.9	0.0	0.0	
Joy	2.9	1.3	1.7	3.8	79.5	0.4		0.5	0.0	0.0	0.0	98.1	0.0	
Sadness	8.0	5.8	5.8	8.0	1.8	56.7		3.4	2.1	0.4	3.8	0.0	87.6	
**Low Dynamism**														
**Schizophrenia Group (n=56)**								**Control Group (n=56)**						
	**Surprise**	**Fear**	**Anger**	**Disgust**	**Joy**	**Sadness**		**Surprise**	**Fear**	**Anger**	**Disgust**	**Joy**	**Sadness**	
Surprise	88.7	4.1	0.0	0.9	2.3	2.3		95.8	3.7	0.0	0.0	0.0	0.0	
Fear	34.2	38.9	1.7	3.8	0.9	12.4		13.1	73.4	0.0	0.4	0.0	12.7	
Anger	1.3	3.4	82.3	5.6	0.4	2.6		0.9	1.8	91.9	4.1	0.0	1.4	
Disgust	0.4	1.3	41.1	47.8	0.4	4.5		1.7	0.9	14.7	82.3	0.0	0.0	
Joy	6.7	1.0	3.3	2.9	73.7	2.4		1.3	0.0	0.0	0.0	96.2	0.0	
Sadness	5.4	10.7	4.5	5.4	0.9	63.8		1.9	7.0	0.5	3.3	0.9	82.7	

Notes: Columns: emotions recognized. Rows: emotions presented.

**Table 5:** Confusion matrix of emotion recognition (%) using frontal and profile views of the DVFs for the schizophrenia group.

**Frontal Views**														
**Schizophrenia Group (n=56)**								**Control Group (n=56)**						
	Neutral	Surprise	Fear	Anger	Disgust	Joy	Sadness	Neutral	Surprise	Fear	Anger	Disgust	Joy	Sadness
Neutral	88.4	1.8	0.0	1.8	0.0	5.4	2.7	98.2	0.0	0.0	0.0	0.0	0.0	1.8
Surprise	1.8	89.3	4.9	0.4	0.4	1.8	1.3	0.4	91.0	8.5	0.0	0.0	0.0	0.0
Fear	7.1	30.4	50.4	1.3	3.1	0.9	6.7	0.9	13.7	75.7	0.4	0.9	0.0	8.4
Anger	2.7	2.2	3.1	83.0	6.3	0.9	1.8	0.4	0.9	1.3	93.8	2.2	0.0	1.3
Disgust	4.0	2.2	4.5	26.3	59.4	0.4	3.1	0.4	0.4	1.8	8.5	88.8	0.0	0.0
Joy	9.8	4.5	1.3	1.8	3.1	78.6	0.9	1.8	0.9	0.0	0.0	0.0	97.2	0.0
Sadness	10.3	7.1	7.6	6.7	6.7	2.2	59.4	2.2	1.8	3.1	0.9	3.6	0.9	87.6
**Profile Views**														
**Schizophrenia Group (n=56)**								**Control Group (n=56)**						
	Neutral	Surprise	Fear	Anger	Disgust	Joy	Sadness	Neutral	Surprise	Fear	Anger	Disgust	Joy	Sadness
Neutral	83.9	0.0	4.5	1.8	3.6	1.8	4.5	96.4	0.0	0.0	0.0	1.8	0.0	1.8
Surprise	2.2	86.2	5.4	0.4	1.8	2.7	1.3	0.4	91.6	7.6	0.0	0.4	0.0	0.0
Fear	4.0	42.0	40.6	0.9	3.1	0.4	8.9	0.5	15.3	77.5	0.0	0.5	0.0	6.3
Anger	4.0	0.9	3.1	83.5	4.0	0.4	4.0	0.5	0.5	1.4	94.6	2.7	0.0	0.5
Disgust	4.9	2.2	3.1	21.4	63.4	0.4	4.5	0.4	1.8	1.3	8.5	87.9	0.0	0.0
Joy	10.7	4.9	0.9	3.1	3.6	75.0	1.8	2.2	0.9	0.0	0.0	0.0	97.0	0.0
Sadness	12.9	6.3	8.9	3.6	6.7	0.4	61.2	4.0	3.6	5.8	0.0	3.6	0.0	83.0

Notes: Columns: emotions recognized. Rows: emotions presented.

## Data Availability

The data presented in this study are openly available in RUIdeRA (hdl.handle.net/10578/27021, accessed on 20 April 2021).
